# CRISPR/Cas9-mediated genome editing in nonhuman primates

**DOI:** 10.1242/dmm.039982

**Published:** 2019-10-01

**Authors:** Yu Kang, Chu Chu, Fang Wang, Yuyu Niu

**Affiliations:** 1 Faculty of Environmental Science and Engineering, Kunming University of Science and Technology, Kunming, Yunnan 650500, China; 2 Institute of Primate Translational Medicine, Kunming University of Science and Technology, Kunming, Yunnan 650500, China; 3 Yunnan Key Laboratory of Primate Biomedicine Research, Kunming, Yunnan 650223, China

**Keywords:** Genome editing, CRISPR/Cas9, Nonhuman primates

## Abstract

Owing to their high similarity to humans, non-human primates (NHPs) provide an exceedingly suitable model for the study of human disease. In this Review, we summarize the history of transgenic NHP models and the progress of CRISPR/Cas9-mediated genome editing in NHPs, from the first proof-of-principle green fluorescent protein-expressing monkeys to sophisticated NHP models of human neurodegenerative disease that accurately phenocopy several complex disease features. We discuss not only the breakthroughs and advantages, but also the potential shortcomings of the application of the CRISPR/Cas9 system to NHPs that have emerged from the expanded understanding of this technology in recent years. Although off-target and mosaic mutations are the main concerns in CRISPR/Cas9-mediated NHP modeling, recent progress in genome editing techniques make it likely that these technical limitations will be overcome soon, bringing excellent prospects to human disease studies.

## Abstract (precis)

**Summary:** This Review discusses the history and development of genome editing in non-human primates, as well as the challenges and prospects facing this technology now and in the future.

## Introduction

**Fig. 1.**
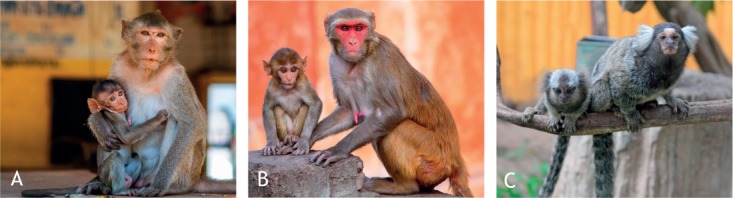
**Three main NHP species most commonly used as human disease models.** (A) *Macaca fascicularis* (crab-eating macaque or cynomolgus). (B) *Macaca mulatta* (rhesus monkey). (C) *Callithrix jacchus* (common marmosets). Images courtesy of Elena Yurkina (A), donyanedomam (B) and Nattapong Jaichansugkit (C). These images are not published under the terms of the CC-BY licence of this article. For permission to reuse, please see www.123RF.com.

Laboratory animals have long played a useful role in biomedical and medical research. Although the mouse has been widely used as the most popular experimental animal, nonhuman primates (NHPs) have many advantages over any other species as a prime model system for the study of human diseases. NHPs feature high similarity to humans in many properties, including genetics, physiology, developmental biology, social behaviors and cognitive ability. DNA sequence similarity between NHPs and humans can be as high as 98.77% (Fujiyama et al., 2002) compared to ∼90% for that of the rodent. Therefore, NHPs are a prime choice for investigating pervasive human health conditions such as cardiac defects and diabetes, as well as infectious, liver and neurodegenerative diseases. Importantly, similarities to humans in the structure and function of the brain make NHPs a unique model for research into human brain disorders. The three main NHPs most commonly used as human disease models include *Macaca fascicularis* (crab-eating macaque or cynomolgus), *Macaca mulatta* (rhesus monkey) and *Callithrix jacchus* (common marmoset) (Fig. 1).

Traditionally, methods for disease modeling in NHPs have included the observation of spontaneously occurring disease, chemical (drug) induction and surgical procedures. In recent years, methods that utilize precise gene modification have been widely applied. Here, we summarize the history of such transgenic models, the progress of NHP genome editing using the clustered regularly interspaced short palindromic repeat (CRISPR)-associated nuclease Cas9 (CRISPR/Cas9) system, and the most recent developments of this technology's application in NHPs.

## Early transgenic models

**Fig. 2.**
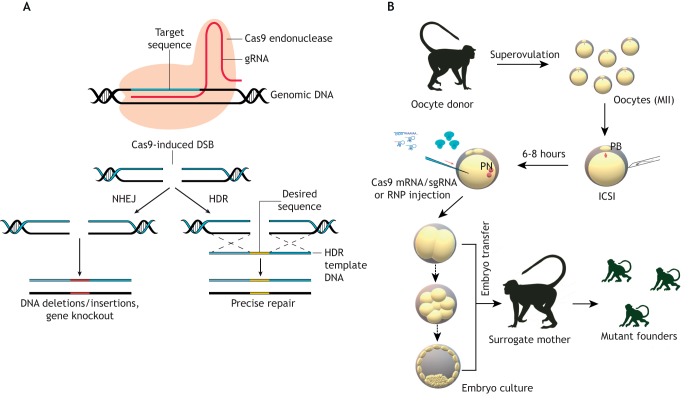
**Schematic of the experimental approach to generate gene-edited monkeys via CRISPR/Cas9.** (A) Schematic of the CRISPR/Cas9 system. The gRNA tethers the Cas9 endonuclease to a specific genetic locus in a sequence-specific manner. The Cas9-induced DSBs are then repaired by intrinsic DNA repair mechanisms. Generally, the simpler error-prone NHEJ results in an indel or frameshift mutation that inactivates the gene. If a repair template is provided, the more precise HDR system can result in a specific alteration, such as a disease-causing point mutation. (B) Genome editing in NHPs. The first step requires harvesting and fertilization of oocytes, which can then be edited via injection of CRISPR components (either Cas9 and sgRNA RNPs, or Cas9 mRNA and sgRNA molecules). The edited embryos are then cultured and implanted in a surrogate mother. dsDNA, double-stranded DNA; ICSI, intracytoplasmic sperm injection; MII, metaphase II stage; PB, polar body; PN, pronucleus; RNP, ribonucleoproteins.

Genome editing is a type of genetic engineering in which DNA is inserted, deleted, modified or replaced in the genome of cells or organisms. This technique can be used, for example, to modify genetic information within an organism's genome and introduce new characteristics, or to remove specific regions of genomes such as those that confer disease susceptibility. In addition, genome editing can be used to add exogenous genes to specific locations in genomes. Viral vectors were the earliest tools used by molecular biologists to deliver genetic material into cells. In the 1970s, Paul Berg and coworkers first used a modified simian virus 40 (SV40) to infect monkey kidney cells maintained *in vitro* (Goff and Berg, 1976). Since those seminal early experiments, the main types of viruses used as genetic vectors have included retroviruses (Doi and Takeuchi, 2015), lentiviruses (Joglekar and Sandoval, 2017) and adenoviruses (Tatsis and Ertl, 2004). Retroviruses contain a reverse transcriptase that synthesizes a DNA copy of the introduced RNA genome and integrates it into the host genome. Lentiviruses are a subclass of retroviruses that can achieve genome integration in non-dividing cells. Another way of gene delivery is through adenoviral vectors, the DNA of which does not integrate into the host genome. However, because adenoviruses cause only a transient infection, continuous injections may be necessary, which can lead to unsatisfactory results owing to a strong immune response in the subject. To overcome these problems, scientists have developed adeno-associated virus (AAV) vectors, which can infect both dividing and non-dividing cells without incorporation into the chromosome for a long time and achieve stable expression with limited immune response. Moreover, after packaging, the size of AAV genomes does not exceed ∼5 kb, which is easily delivered into the host cell. These features have made AAV a very attractive candidate as a viral vector for gene therapy. In a recent study, Wang et al. (2018a) used a single infusion of AAV to deliver the CRISPR/Cas9 components (Fig. 2A) targeting *PCSK9* in the liver. PCSK9, an antagonist of the low-density lipoprotein (LDL) receptor, induces hypercholesterolemia if highly expressed. The authors showed that the six NHPs with *PCSK9* knocked out had a normal concentration of serum LDL. This research highlighted safety considerations for gene therapy of hypercholesterolemia (Wang et al., 2018a). In addition, the relatively low genotoxicity profile of recombinant AAV (rAAVs) provides an advantage. Wang et al. (2019) delivered rAAV-packaged CRISPR/Cas9 components to NHP embryos, achieving robust gene editing efficiency.

Back in 2001, researchers used genome editing to produce the first transgenic animal in the USA: a transgenic monkey was born containing the green fluorescent protein (GFP) transgene, which was introduced by injecting a replication-defective retroviral vector carrying this gene into the perivitelline space of rhesus monkey oocytes (Chan et al., 2001). In the following years, researchers used different lentiviral vectors to obtain various transgenic monkeys, including human disease models, with high efficiency. For example, in 2008, Yang et al. reported the first transgenic NHP human disease model, a rhesus macaque that expressed polyglutamine-expanded *HTT* which would lead to Huntington's disease. The animal showed both genetic modification and the associated neurodegenerative phenotypes (Yang et al., 2008). In 2009, Sasaki and colleagues generated a transgenic marmoset using a self-inactivating lentiviral vector in which transgenes could be passed onto the next generation (Sasaki et al., 2009). Furthermore, in 2010, our group reported the usefulness of simian immunodeficiency virus (SIV)-based lentiviral vectors for the generation of transgenic monkeys and developed a protocol for infection of early-cleavage-stage embryos (Niu et al., 2010).

## Precise genome editing systems

The major drawback of lentivirus-based gene editing is the possibility of random insertion into the host genome. As a result of this uncertainty, non-targeted loci can be influenced by the insertion and lead to unexpected phenotypical consequences, regardless of whether they insert in coding or non-coding regulatory regions. Therefore, genetic models of some diseases, especially those caused by specific mutations, have been difficult to generate. Besides, it is often necessary to disrupt a target gene or artificially replace a gene sequence in order to analyze the functions of the genes of interest and their relevance to diseases in detail (Sato and Sasaki, 2018). Thanks to the emergence of innovative genome editing technologies, precise modification of NHP genomes became feasible. The most prominent technologies for precise genome editing include zinc-finger nucleases (ZFNs), transcription activator-like effector nucleases (TALENs) and CRISPR/Cas9. ZFNs and TALENs have the ability to recognize virtually any sequence by customized DNA-binding nucleases, although building these custom proteins requires a lot of manpower and material resources. However, the arrival of the CRISPR/Cas9 system, a bacterial protection mechanism against phages, largely solved these issues.

There are three main types of CRISPR systems, among which those belonging to the type II are most commonly used in gene editing applications. One such type II system is CRISPR/Cas9, which mediates genome editing via Watson-Crick complementary base pairing. A single guide RNA (sgRNA) recognizes a target DNA sequence and guides the endonuclease component, Cas9, which cuts the DNA. In more detail, the working mechanism of this system can be split into the following steps. First, long primary pre-CRISPR RNA (pre-crRNA) is transcribed from the CRISPR loci in bacteria, then pre-crRNA pairs with trans-activating crRNA (tracrRNA) to be processed by RNase III. Simultaneously, Cas9 catalyzes the formation of a crRNA-tracrRNA complex, followed by the generation of shorter mature crRNA. Finally, this mature crRNA and the tracrRNA form a double-stranded RNA structure that directs Cas9 towards a target locus where it generates double strand breaks (DSBs) in the DNA (Ran et al., 2013). This system has been adapted to work in eukaryotes by supplying extrinsic gRNA sequences instead of relying on transcription from endogenous CRISPR loci. In addition, researchers have modified the catalytic domains of Cas9 to either induce DSBs or single-stranded nicks if the application requires this type of cut. In response to the DSBs, intrinsic cellular DNA repair processes mostly result in random insertions or deletions (indels) at the site of DNA cleavage through non-homologous end joining (NHEJ) (Fig. 2A).

The development of this precise gene editing system that does not require customized protein synthesis for targeted nuclease activity offered a new and elegant way to generate animal models of genetic defects, enabling detailed characterization of the functional consequences of specific genetic changes. Furthermore, precise genome modification technologies allow efficient generation of isogenic cell lines with and without disease mutations, which can then be differentiated into somatic cell types for disease or cell therapy research. Until now, precise genetic engineering in mammalian species has been most common and successful in mice, whereby the main associated methods include zygote-based microinjection approaches for transgenesis and gene knockout (KO)/knock-in (KI) approaches using embryonic stem cells (Gurumurthy and Lloyd, 2019).

## Gene disruption in NHPs by CRISPR/Cas9

Several researchers edited the genomes of NHPs with CRISPR/Cas9, as summarized in Table 1. For example, in 2014, our group generated KO cynomolgus monkeys by targeting one-cell embryos with CRISPR/Cas9 (Fig. 2B), which for the first time demonstrated the feasibility of this genome editing system in NHPs (Niu et al., 2014). In that study, we modified three genes, *Nr0b1* (also known as *DAX1*), *Ppar-γ* (*Pparg*) and *Rag1*, with targeting efficiencies of 26.6%, 46.7% and 60% in the embryo, respectively. Similarly, in 2014, Wan et al. generated a *Tp53* biallelic mutant cynomolgus monkey (Wan et al., 2015). Their results showed that biallelic gene mutation could be efficiently generated in monkeys by optimizing the sgRNA sequence and the Cas9-mRNA/sgRNA concentrations. Because of the long sexual maturation time and low reproduction rate, generating biallelic mutant NHPs for loss-of-function studies through breeding is very challenging. Nevertheless, the biallelic/homozygous mutant monkeys produced by this method will help to bring genetically modified NHP models closer to practical application. In the same year, two reports showed the possibility of using the CRISPR/Cas9 system to generate NHP models for human disease. Chen et al. used this system to disrupt the monkey dystrophin gene (*DMD*), mutations of which cause Duchenne muscular dystrophy (DMD) (Chen et al., 2015). The newborn Cas9-targeted monkeys showed muscle degeneration, suggesting that the loss of dystrophin could affect the development of muscle cells at a very early stage. In the second study, Kang et al. described a male fetal monkey in which CRISPR/Cas9 genome editing had produced *Nr0b1*-null mutations in most somatic tissues and in the gonads, successfully recapitulating human adrenal hypoplasia congenita and hypogonadotropic hypogonadism (AHC-HH) (Kang et al., 2015). Two years later, Midic and colleagues used the β-hemoglobin (*HBB*) gene as the test locus for CRISPR/Cas9 to assess the targeting efficiencies, specificities, timing and genetic mosaicism outcomes in rhesus monkeys, and determined a high targeting efficiency (80-100%) in embryos (Midic et al., 2017). In 2018, Zhang et al. published their research on the roles of *SIRT6* in primates using precise genome editing (Zhang et al., 2018). In a one-step procedure using CRISPR/Cas9, they generated a biallelic SIRT6-null cynomolgus monkey model that exhibited severe prenatal developmental growth retardation and died hours after birth. To explore the role of *SIRT6*, the authors conducted a series of functional experiments, which overall indicated that SIRT6 acts as a mediator of primate brain development by repressing the H19 long non-coding RNA in a *trans* manner. This study opened a new avenue for modeling and studying the pathogenesis of human perinatal lethality syndrome. Although precise gene modification in marmosets with ZFNs was reported in 2016 (Sato et al., 2016), gene targeting using CRISPR/Cas9 was first reported in February 2019, in a study describing successful gene KI in marmoset embryonic stem cells and early embryos (Yoshimatsu et al., 2019). However, a CRISPR/Cas9-mediated gene KO marmoset model was only described in September 2019 (Kumita et al., 2019).

**Table 1.**
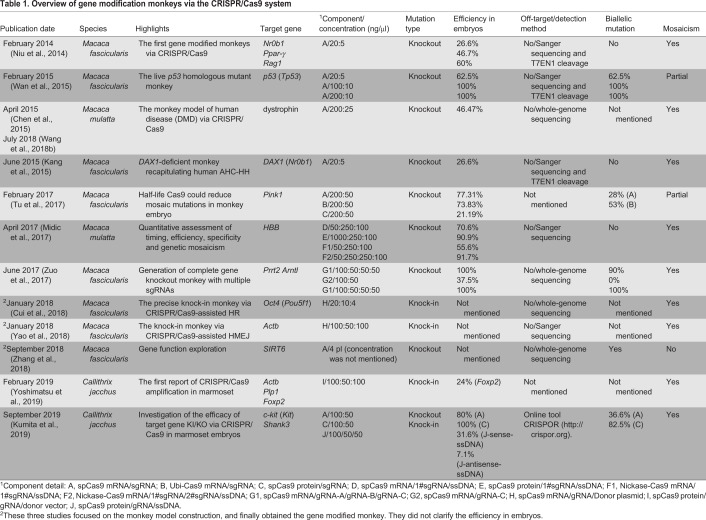
**Overview of gene modification monkeys via the CRISPR/Cas9 system**

## Gene introduction into NHPs by CRISPR/Cas9

At a target genomic site, the CRISPR/Cas9 system can accurately and effectively introduce a DSB that triggers two basic repair systems. If this DNA damage is repaired through NHEJ, it will likely result in a target gene KO with random indels. Conversely, if the DSB is repaired by homology-directed repair (HDR), a desired fragment could be introduced into the target site as long as the donor template DNA that harbors a homologous sequence corresponding to the genomic DNA around the DSB site is provided (Fig. 2A). Precise gene KI allows researchers to introduce the desired phenotype and to understand the function of the regulatory region of a target gene. In addition, the ability to replace the mutant sequence with the correct one is the foundation of gene therapy for genetic disease (Yoshimatsu et al., 2019). As homologous recombination (HR) can only occur during a short part (late S/G2 phases) of the cell cycle, the efficiency of correct HDR-mediated gene KI (10% on average) is much lower than that of KO (up to 100%). HDR-mediated KIs have been generated in many species since 2014 (Johnson et al., 2016; Wu et al., 2016; Yang et al., 2016). However, possibly owing to the complexity of DNA repair mechanisms (Luo et al., 2016), KI by CRISPR/Cas9 in NHPs was only achieved in January 2018, when our lab, concurrently with the Huang and Yang groups, reported KI cynomolgus monkeys with a reporter gene generated via CRISPR/Cas9-assisted HR and homology-mediated end joining (HMEJ) (Cui et al., 2018; Yao et al., 2018). Sasaki and colleagues reported precise gene KI marmoset models in 2019 (Kumita et al., 2019; Yoshimatsu et al., 2019). These results confirm the feasibility of generating NHP models of human disease by introducing disease-causing gene variants using the CRISPR/Cas9 system.

## Off-target effect of CRISPR/Cas9 in NHPs

Influencing non-targeted genes (the off-target effect) has been a controversial issue since the first reports of the CRISPR/Cas9 system. A significant number of experiments revealed undesired cleavage by Cas9 at off-target genome sites at which the DNA sequence was partly homologous (with one or more mismatches) to the 20-base sgRNA. The specificity of the CRISPR/Cas9 system mainly depends on the number of homologous off-target sites. Thus, for species with large genomes such as humans and NHPs, avoiding off-target Cas9 activity is the major challenge in the application of this technology. Furthermore, it is critical for clinical safety that all off-target effects are eliminated when the system is used as a therapeutic reagent. Many research groups have focused efforts on reducing this phenomenon. For example, scientists have designed mutant Cas9 nucleases with higher specificities than that of the wild-type protein, such as enhanced specificity Cas9 (eSpCas9) (Slaymaker et al., 2016), Cas9-high fidelity (Cas9-HF) (Kleinstiver et al., 2016) and Sniper-Cas9 (Lee et al., 2018). Meanwhile, other groups have focused on the optimization of gRNAs. For example, Fu et al. showed that sgRNAs with 5′-GG- can greatly reduce off-target cutting, and that using truncated gRNAs can also improve CRISPR/Cas9-induced DSB specificity (Fu et al., 2014). Other researchers have contributed various advanced methods for the detection of off-target genome editing, including deep sequencing (Anderson et al., 2018), web-based prediction tools (Bae et al., 2014) and ChIP-seq (O'Geen et al., 2015), all of which have been widely adopted.

Most recently, Pinar and coworkers described a new method called ‘verification of *in vivo* off-targets’ (VIVO), a highly sensitive two-step strategy that can robustly identify genome-wide off-target effects of Cas nucleases (Akcakaya et al., 2018). In this study, before conducting the *in vivo* genome editing experiment, the researchers used the circularization for *in vitro* reporting of cleavage effects using a sequencing approach (CIRCLE-seq) to discover off-target cleavage sites. They then screened the genomes of edited animals for these potential off-target mutations. This technique was shown to be highly sensitive, avoided potential confounding effects associated with cell-based off-target screening assays and could successfully identify supersets of sites that include bona fide off-targets in cultured human cells. Although several approaches can identify off-target sites, VIVO is, up to now, the most reliable method. Fortunately, the research on gene modification by CRISPR/Cas9 in NHPs described above and in Table 1 has indicated no off-target effects, although researchers used a number of detection techniques in their individual studies. For example, earlier in 2018, Wang et al. used whole-genome sequencing to comprehensively assess on- and off-target mutations in NHPs in which a genetic disorder mimicking DMD was introduced by CRISPR/Cas9-based gene editing. The authors found that the system targeted the expected genomic sites without producing off-target modifications in other functional regions of the genome (Wang et al., 2018b).

Although all the results of gene editing in NHPs appear to be positive thus far, more attention should still be devoted to identifying and minimizing potential off-target effects in NHPs, especially considering the complexity of their genome, their significance for human disease research and the specific ethical considerations for research in these species.

## Mosaic mutations introduced by CRISPR/Cas9

In addition to off-target effects, genetic mosaicism, in which cells within an organism have different genomes, is another issue that may hinder the application of genome editing for disease modeling, as components of the CRISPR/Cas9 system can repeatedly edit target genes at different stages of embryonic development. This repeated editing induces problems in two ways: the founding animals may carry both mutant and wild-type alleles, and different mutations may be induced in different cells of an animal. Both of these effects lead to genetic mosaicism. Owing to the long time for sex maturation (4-5 years) and limited reproductive capacity (1-2 offspring per year), it is especially important to avoid mosaicism in genetically modified NHPs.

The prolonged expression of Cas9 mRNA was thought to be a potential mosaicism-inducing element. However, direct injection of the Cas9 protein into cells also leads to mosaic mutations (Kim et al., 2014). For example, Li and colleagues believe that the prolonged existence and activity of Cas9 in embryos will contribute to mosaicism even though the precise mechanisms underlying CRISPR/Cas9-mediated mosaic mutations remain elusive. In 2017, the group published their research on the reduction of mosaic mutations in NHP embryos through shortening the half-life of Cas9, which they achieved by tagging the protein with ubiquitin-proteasomal degradation signals (Tu et al., 2017). In the same year, Yang and colleagues tried to eliminate mosaicism by completely knocking out the target gene (Zuo et al., 2017). The researchers designed multiple adjacent sgRNAs (spaced 10-200 bp apart) to target a single key exon of each gene and co-injected the sgRNAs with Cas9 mRNA into zygotes of mouse and monkey. In the monkey, they achieved a complete gene KO with high efficiencies (100% for *Arntl* and 91% for *Prrt2*) in the embryos and obtained a complete *Prrt2* KO monkey in a single step. Importantly, they performed whole-genome sequencing on the positive samples at a sufficient depth to detect off-target mutations and found no indels among the 1947 potential off-target sites. The above results indicate that it is possible to obtain single homozygous mutations in monkey, thus avoiding a mosaic genotype.

Overall, advances in the CRISPR/Cas9 genome editing technique have made it possible to generate genetically modified NHPs, which provide a new and exciting way to explore gene functions, disease mechanisms and human therapy strategies in modern translational medical research. As mentioned above, almost all the studies revealed that CRISPR/Cas9 creates mosaic mutations in NHP embryos, which has also recently been seen in non-viable human embryos derived from human tripronuclear zygotes injected with CRISPR/Cas9 (Liang et al., 2015; Tang et al., 2017). Because of the safety concerns associated with using CRISPR/Cas9 in human embryos and the serious ethical issues involved in human germline modification, it is important to further investigate the use of CRISPR/Cas9 system in NHP embryos to generate faithful animal models of human disease and provide a robust therapeutic platform.

## Conclusion

One of the ultimate goals of translational medical research is to use experimental animals to substitute the human body to learn how an illness develops, progresses and affects the organism. In addition, all potential disease treatments, such as drugs, vaccines or technologies, must pass the animal experiment phase before any clinical application. Overall, all efforts are made to improve human health and lengthen the lifespan of humans, while simultaneously ensuring animal welfare. We are mindful of the ethical considerations and are adopting the appropriate 3R (replacement, reduction and refinement) guidelines. As species that are closely related to humans, NHPs bring great hope for improving our understanding of human diseases and finding the appropriate treatment strategy. It is also worth mentioning that commonly used NHP species in biomedical research (Fig. 1) are smaller and evolutionarily less close to humans than, for example, chimpanzees and bonobos. The development of genome editing technologies, in particular the CRISPR/Cas9 system, has enabled invaluable progress in the generation of NHP models of human diseases that can help us understand disease in ways that small-animal models cannot. In only a few years since the discovery of CRISPR/Cas9, this fast-growing technology has brought both high hopes and challenges to translational medical research, including the important conversations on safety and ethical concerns associated with it.

Nowadays, the CRISPR/Cas9 system is being constantly improved. Besides the evolution of the technology itself, CRISPR/Cas9 has also been combined with other methodologies, such as genetically modified stem cells for cell therapy, chimeric antigen receptor T cells (CAR-T) for cancer therapy, and somatic cell nuclear transfer (SCNT) for generating disease models, with the potential to bring groundbreaking progress to modern biomedical research. In an example of research enabled by the CRISPR/Cas9-mediated genome editing system, breakthroughs in NHP SCNT research (Liu et al., 2018), the technology of monkey cloning, could make a significant contribution to disease research in physiologically relevant models.

Although a human disease model could be generated by direct injection of CRISPR/Cas9 components into zygotes, several problems still exist, including mosaicism (Yen et al., 2014), random mutations and the relatively low editing efficiency for HDR-mediated KI or KO. These issues are perhaps less significant in small-animal models with their relatively short generation times and large litter sizes, but could render this approach infeasible for large animals such as pigs, sheep or monkeys, which have longer gestation periods, sexual maturation times and uniparous features. Taking the cynomolgus monkey as an example, gestation takes 155 to 160 days to produce a single offspring per pregnancy, and an additional 3-4 years for sexual maturation. Unlike cynomolgus, rhesus monkeys are seasonal breeders, which means they generate offspring during a certain time of the year (Muehlenbein et al., 2002). Although the common marmosets have a shorter gestation period (144 days), and normally have twins, it still takes much longer to get the next generation than in smaller animals such as rodents. To compensate for this, somatic cells with specific mutations could be produced by CRISPR/Cas9 and screened *in vitro* and, when combined with the SCNT procedure, a genetically defined monkey model could be generated within 1-2 years. Although the efficiency of a cloned monkey could be increased to 33.3% (live birth) by the injection of a critical histone demethylase of H3K9me3, lysine demethylase 4D (KDM4D), the authors of this study still observed some issues such as post-implantation defects and abnormal placentas (Liu et al., 2018), indicating that there are additional epigenetic barriers that impede SCNT embryo development. Nevertheless, cloning efficiency has been improved in recent research, and such an approach will likely be the most efficient way to generate genetically engineered NHP models of human diseases in the near future.

Overall, the development of genome editing has enabled the generation of NHP and other large animal models, such as pig (Chuang et al., 2017; Perleberg et al., 2018) and sheep (Wang et al., 2016; Williams et al., 2018), to mimic human diseases, but some technical limitations for its application remain to be solved. Thanks to the recent and ongoing breakthroughs and increased awareness of pitfalls and shortcomings, these technology limitations are likely to be overcome soon, allowing the community to make important future progress towards efficient, accurate and accessible tools and models to battle human diseases.
